# Supplementary material for: Structure of a secreted lancelet carotenoid cleavage dioxygenase (CCD), from an ancestral CCD clade
Source: bioRxiv. 2026 Oct 30:2026.10.29.762971. Preprint. [Version 1] doi: 10.64898/2026.10.29.762971 (PMC13734108; doi:10.64898/2026.10.29.762971)
Supplement: Supplementary file 1 [file NIHPP2026.10.29.762971v1-supplement-1.pdf]

bioRxiv preprint doi: https://doi.org/10.64898/2026.10.29.762971; this version posted October 31, 2026. The copyright holder for this preprint (which was not certified by peer review) is the author/funder, who has granted bioRxiv a license to display the preprint in perpetuity. It is made available under a CC-BY 4.0 International license.

Supporting data for:

Crystal structure of a secreted glycosylated lancelet carotenoid cleavage dioxygenase (CCD), an enzyme from an ancestral clade of metazoan CCDs

Sheetal Uppal[1], Gleysin Cabrera Herrera[1], Liya Muslinkina[2], Gowthaman Govindarajan[2], David N. Garboczi[2], Igor B. Rogozin[3†], Eugenia Poliakov[1], Apostolos G. Gittis[2], T. Michael Redmond[1*].

[1]Laboratory of Retinal Cell & Molecular Biology, National Eye Institute, NIH, Bethesda, MD 20892.
[2]Structural Biology Section, Research Technologies Branch, National Institute of Allergy and Infectious Diseases, NIH, Bethesda, MD 20892.
[3]Life Science Research Centre, Faculty of Science, University of Ostrava, Ostrava, 710 00 Czech Republic.

bioRxiv preprint doi: https://doi.org/10.64898/2026.10.29.762971; this version posted October 31, 2026. The copyright holder for this preprint (which was not certified by peer review) is the author/funder, who has granted bioRxiv a license to display the preprint in perpetuity. It is made available under a CC-BY 4.0 International license.

Table S1. **Data collection and refinement statistics. (***Highest resolution shell is shown in parenthesis.**)**

**PDB ID**	**9PLV**
**Data collection**	
Space group	*C2*
Cell dimensions	
a, *b*, *c* (Å)	98.46, 139.91, 93.76
α, β, γ (°)	90, 98.26, 90
Resolution (Å)	24.85 - 2.40 (2.46 - 2.40) *
R_{sym} or R_{merge}	0.089 (0.328)
$I / \sigma I$	11.98 (2.82)
Completeness (%)	98.1 (89.6)
Redundancy	4.25 (3.0)
**Refinement**	
Resolution (Å)	24.56 - 2.40
No. reflections	48,073
R_{work} / R_{free}	0.175/0.224
No. atoms	
Protein	7,629
Ligand/ion/solutes	365
Water	275
B-factors (Å^2)	
Protein	32.99
Ligand/ion/solutes	47.01
Water	34.31
R.m.s deviations	
Bond lengths (Å)	0.008
Bond angles (°)	1.021

bioRxiv preprint doi: https://doi.org/10.64898/2026.10.29.762971; this version posted October 31, 2026. The copyright holder for this preprint (which was not certified by peer review) is the author/funder, who has granted bioRxiv a license to display the preprint in perpetuity. It is made available under a CC-BY 4.0 International license.

Table S2. **Transcript gene counts for BCO proteins in guts of wild type lancelet.**

gene name	Transcripts gene counts			Fixing acidic residues in active site		
	lancelet4	lancelet 7	Lancelet9	**1**	**2**	**3**
LOC118409706 (BCOLg, XP_035666855.1)	432	81	230	**D**	**D**	**E**
LOC118412454 (BCOLb, XP_035671225.1	470	1761	304	**D**	**D**	**E**
LOC118405420 (BCOLd) (XP_035660812.1)	625	952	2049	**D**	**E**	**E**
LOC118405416 (BCOLa1) (XP_035660808.1)	147	386	401	**D**	**E**	**E**
LOC118430142 (BCOLc, XP_035696745.1)	115	154	66	**D**	**D**	**E**
LOC118430143 (BCOLf (XP_035696746.1)	47	78	86	**D**	**E**	**E**
LOC118405340 (BCOa, XP_035660679.1)	3557	13015	3106	**E**	**E**	**E**
LOC118422787 (BCOb, XP_035686436.1)	455	846	343	**E**	**E**	**E**
LOC118426367 (BCOc, XP_035691598.1)	581	2584	2067	**E**	**E**	**E**
LOC118430335 (BCOLe, (XP_035697056.1)	0	0	0	**D**	**E**	**E**

bioRxiv preprint doi: https://doi.org/10.64898/2026.10.29.762971; this version posted October 31, 2026. The copyright holder for this preprint (which was not certified by peer review) is the author/funder, who has granted bioRxiv a license to display the preprint in perpetuity. It is made available under a CC-BY 4.0 International license.

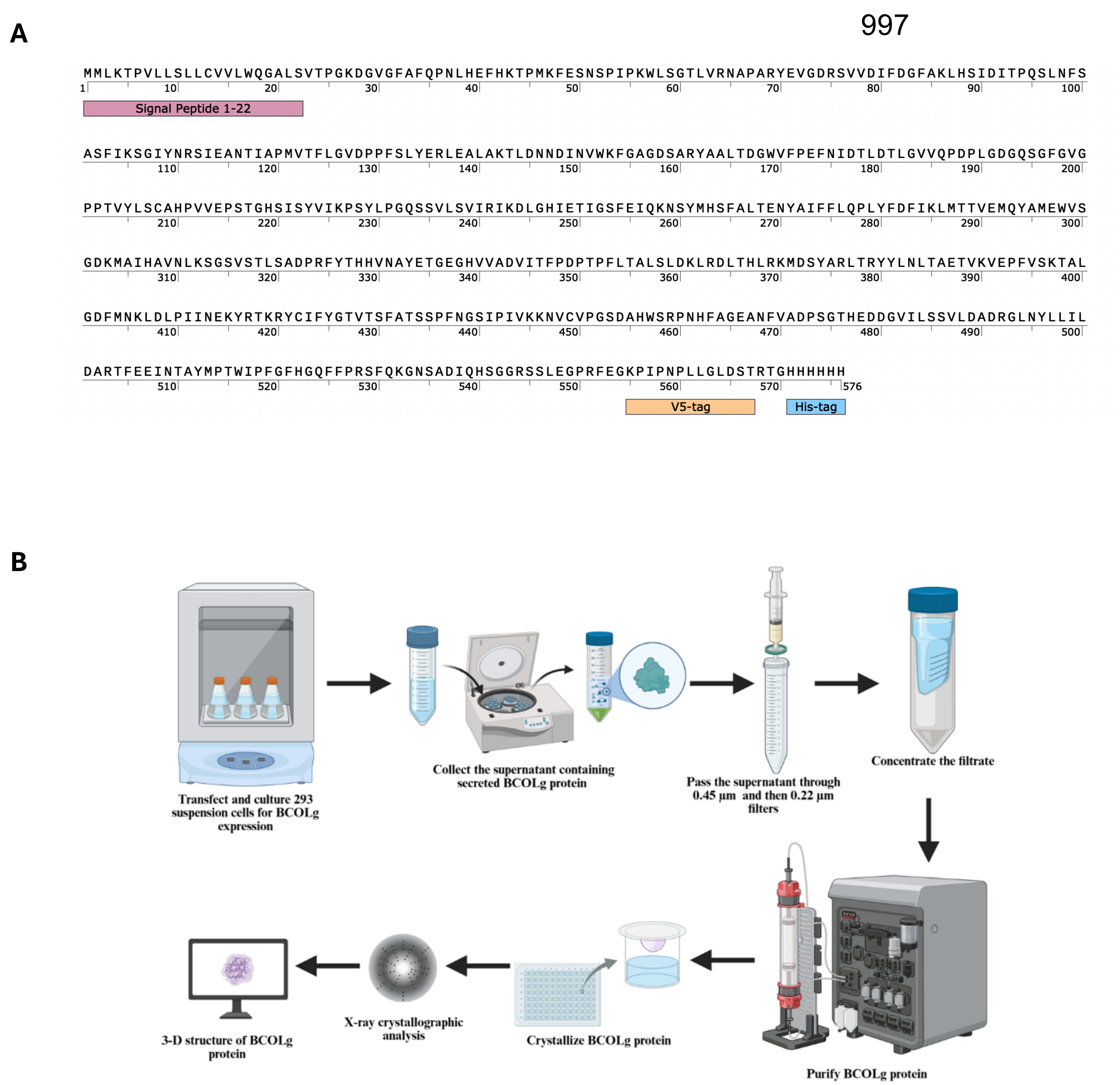

Figure S1. Production of recombinant BCOLg. (A) Schematic of the BCOLg construct cloned into the pcDNA3.1/V5-His-TOPO backbone, showing the signal peptide spanning the amino acid residues 1-22 and the C-terminal V5 and His tags. (B) Workflow for the expression and purification of recombinant BCOLg.

bioRxiv preprint doi: https://doi.org/10.64898/2026.10.29.762971; this version posted October 31, 2026. The copyright holder for this preprint (which was not certified by peer review) is the author/funder, who has granted bioRxiv a license to display the preprint in perpetuity. It is made available under a CC-BY 4.0 International license.

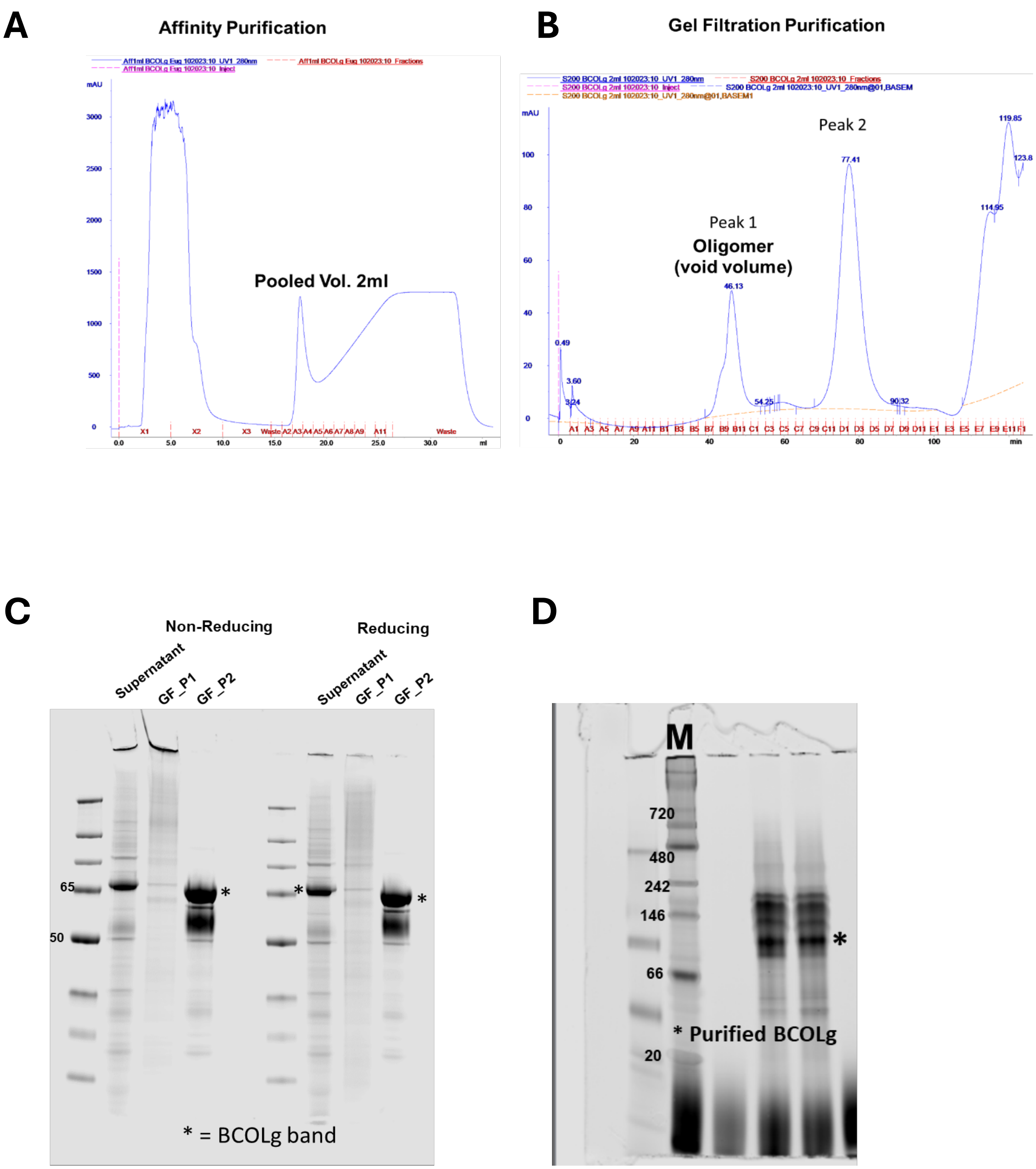

Figure S2. Production of recombinant BCOLg. (A) His-tag TALON (cobalt-based) affinity purification elution profile. (B) Subsequent gel filtration purification elution profile. (C) Noticeable protein degradation in peak 2 eluted from gel filtration column on SDS-PAGE reducing (left panel) and non-reducing (right panel) conditions. (D) Native gel for purified BCOLg showing the band corresponding to the dimer.

bioRxiv preprint doi: https://doi.org/10.64898/2026.10.29.762971; this version posted October 31, 2026. The copyright holder for this preprint (which was not certified by peer review) is the author/funder, who has granted bioRxiv a license to display the preprint in perpetuity. It is made available under a CC-BY 4.0 International license.

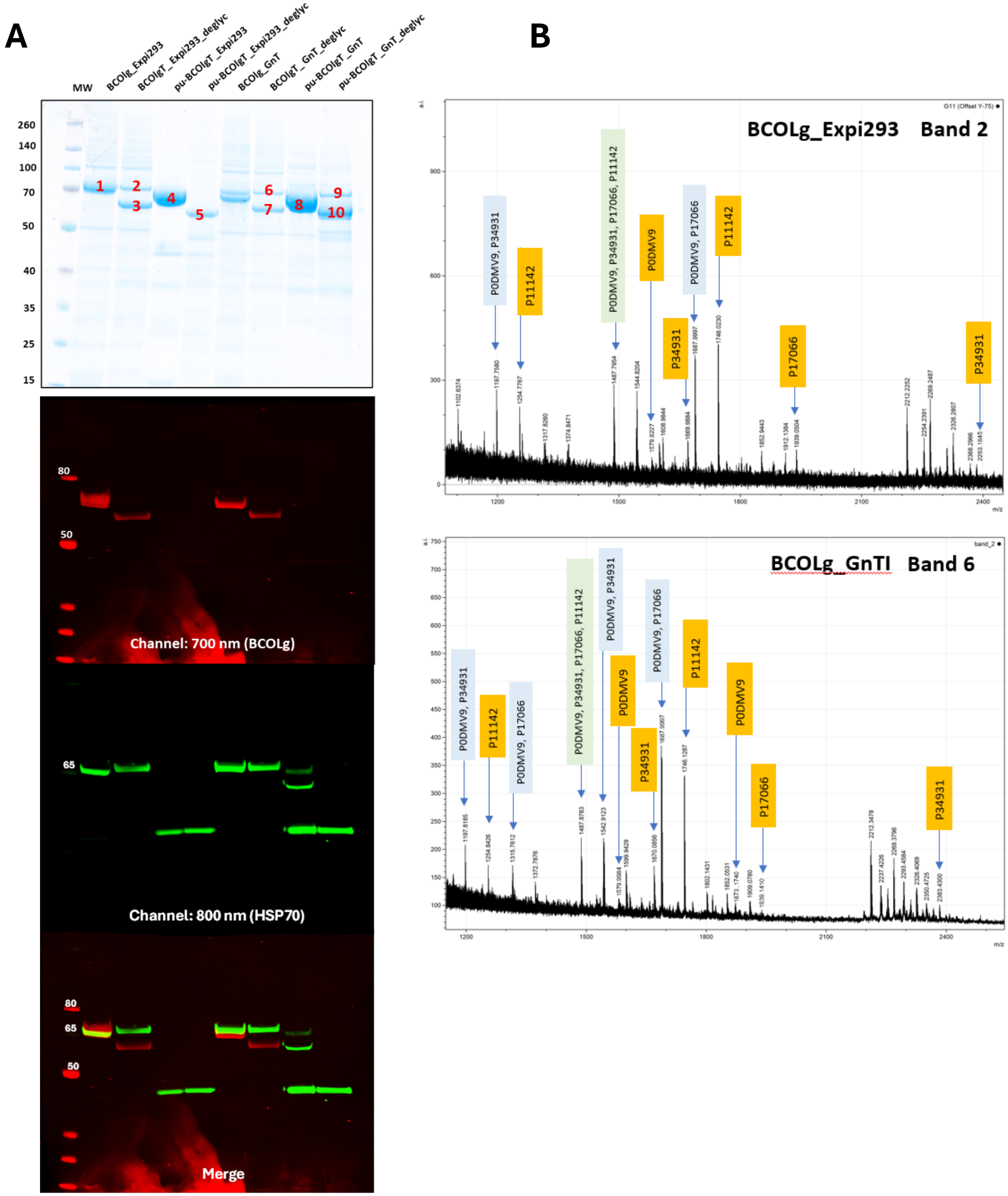

bioRxiv preprint doi: https://doi.org/10.64898/2026.10.29.762971; this version posted October 31, 2026. The copyright holder for this preprint (which was not certified by peer review) is the author/funder, who has granted bioRxiv a license to display the preprint in perpetuity. It is made available under a CC-BY 4.0 International license.

Figure S3. BCOLg is efficiently expressed and purified, and a distinct glycoform-dependent cell line is revealed by SDS-PAGE and mass spectrometry.

(A) Coomassie-stained SDS-PAGE (upper panel) and western blotting (three lower panels) showing BCOLg and HSP70 in cell culture supernatants (Expi293 and GnTI-) and purified BCOLg samples before and after N-deglycosylation by PNGaseF treatment. Bands excised from the gel for additional analysis are numbered (1-10). Purified BCOLg was not identified due to the loss or masking of the C-terminal containing the His6 (Fig. S5) according to MS analysis (data not shown), and HSP70 was identified at a lower mass in comparison with the expected molecular weight, probably related to the degradation process according to results obtained for purified BCOLg from GnTI cells (pu-BCOLg_GnTI-).

(B) MALDI mass spectrometry analysis for identification of contaminant HSP70 isoforms. Representative spectra from HSP70 in Expi293 (Band 2) and GnTI- (Band 6) samples show distinct peptide mass fingerprints, indicating different HSP70 isoforms, as annotated in each mass spectrum. Peptides assigned to HSP70 isoforms are labeled by their protein ID and color (yellow is for unique peptides for this isoform, blue is for peptides matching two proteins, and green for peptides for matching four proteins) PODMV corresponds to HSPA1B, P34931 corresponds to HSPA1L (confirmed by western blotting), P17066 corresponds to HSPA6, P11142 corresponds to HSPA8.

bioRxiv preprint doi: https://doi.org/10.64898/2026.10.29.762971; this version posted October 31, 2026. The copyright holder for this preprint (which was not certified by peer review) is the author/funder, who has granted bioRxiv a license to display the preprint in perpetuity. It is made available under a CC-BY 4.0 International license.

A

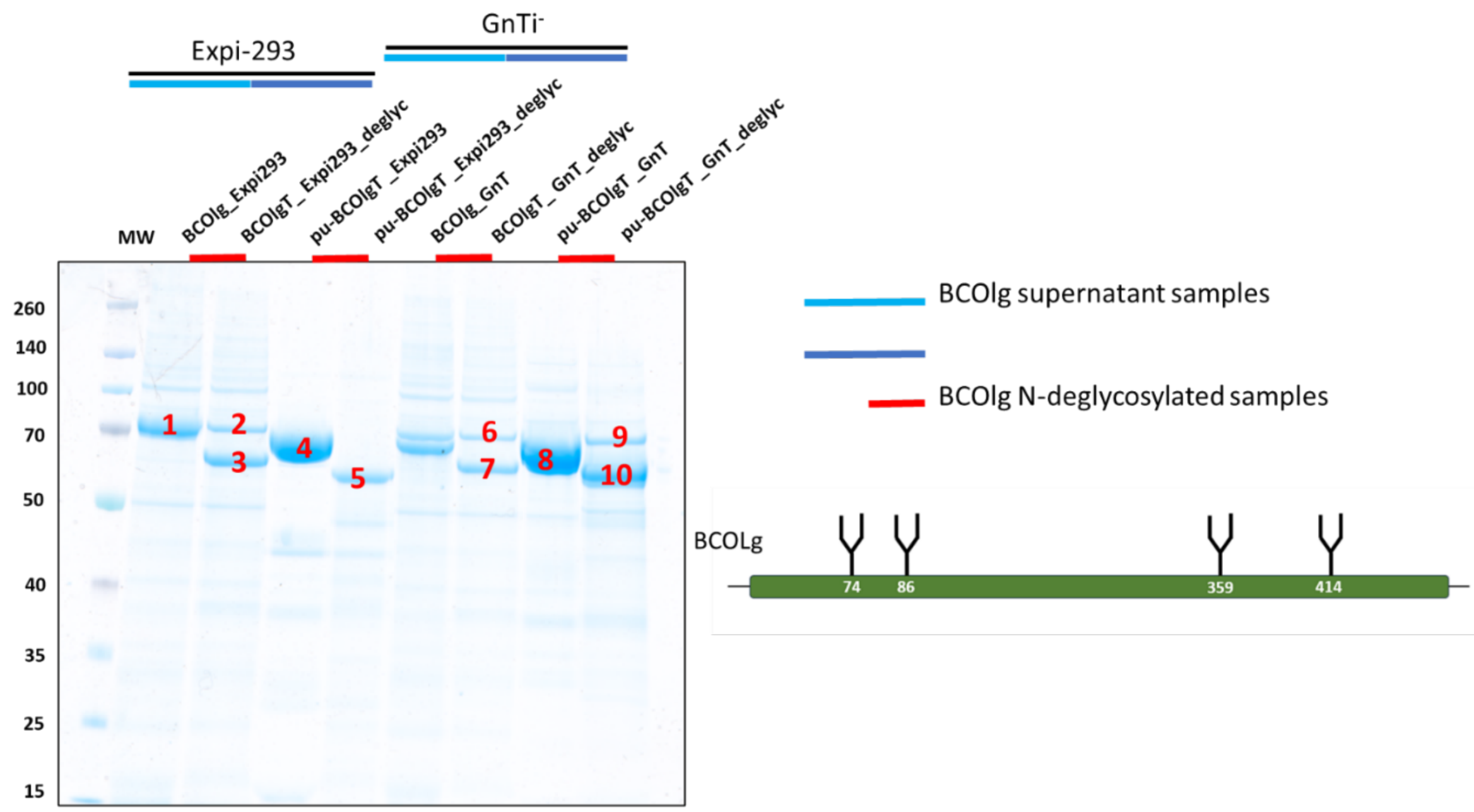

B

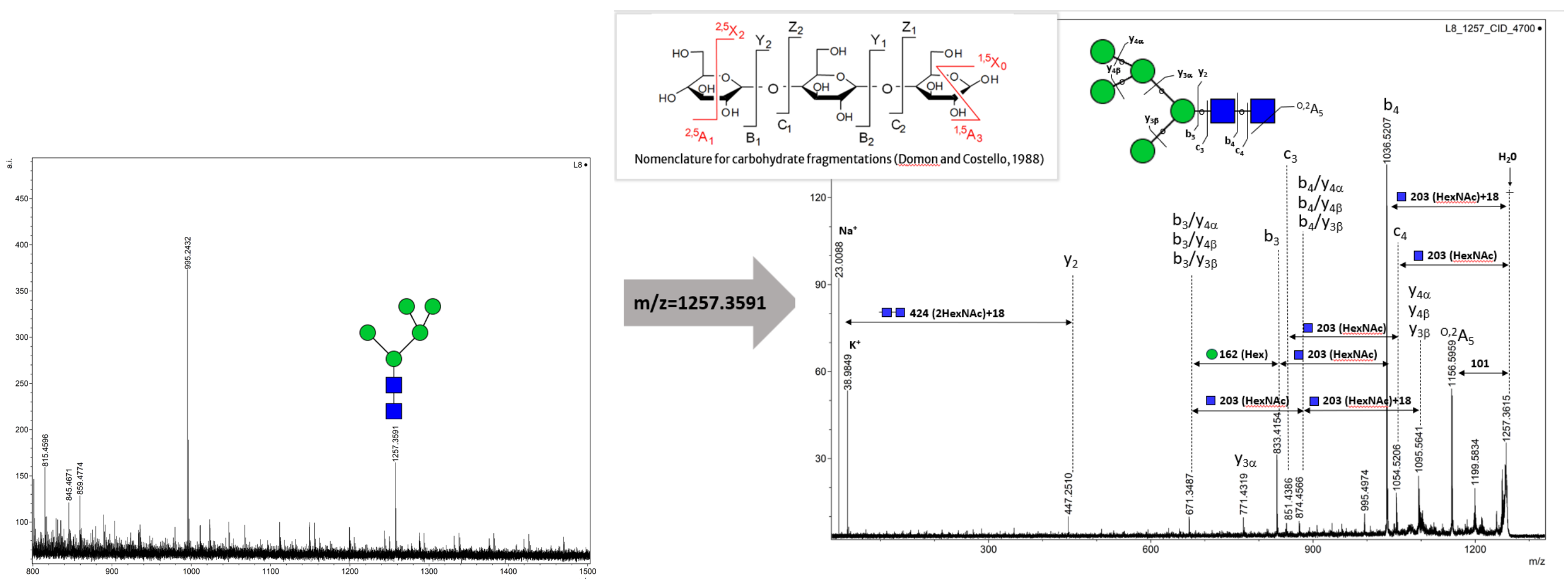

Figure S4. N-glycosylation analysis of BCOLg by gel electrophoresis and mass spectrometry.

(A) Schematic representation of BCOLg indicating four N-linked glycosylation sites (N74, N86, N359, N414). (B) Coomassie-stained SDS-PAGE showing BCOLg in cell culture supernatants (Expi293 and GnT) and purified BCOLg samples before and after N-deglycosylation by PNGaseF treatment. Bands excised from the gel for additional analysis are numbered (1-10). (C) MALDI-MS spectrum of the released N-glycans from band 10. The two higher-intensity signals correspond to micro-cellulose (m/z 955.2432), used in the N-glycan purification protocol, and the oligomannosidic N-glycan structure ($Man_5GlcNAc_2$; m/z 1257.3591), which is the expected N-glycan structure linked to glycoproteins expressed in the GNTI cell line. (D) MALDI-MS/MS spectrum of the signal at m/z 1257.3591 in (C) to confirm the presence of $Man_5GlcNAc_2$ in BCOL expressed in the GNTI cell line.

bioRxiv preprint doi: https://doi.org/10.64898/2026.10.29.762971; this version posted October 31, 2026. The copyright holder for this preprint (which was not certified by peer review) is the author/funder, who has granted bioRxiv a license to display the preprint in perpetuity. It is made available under a CC-BY 4.0 International license.

A

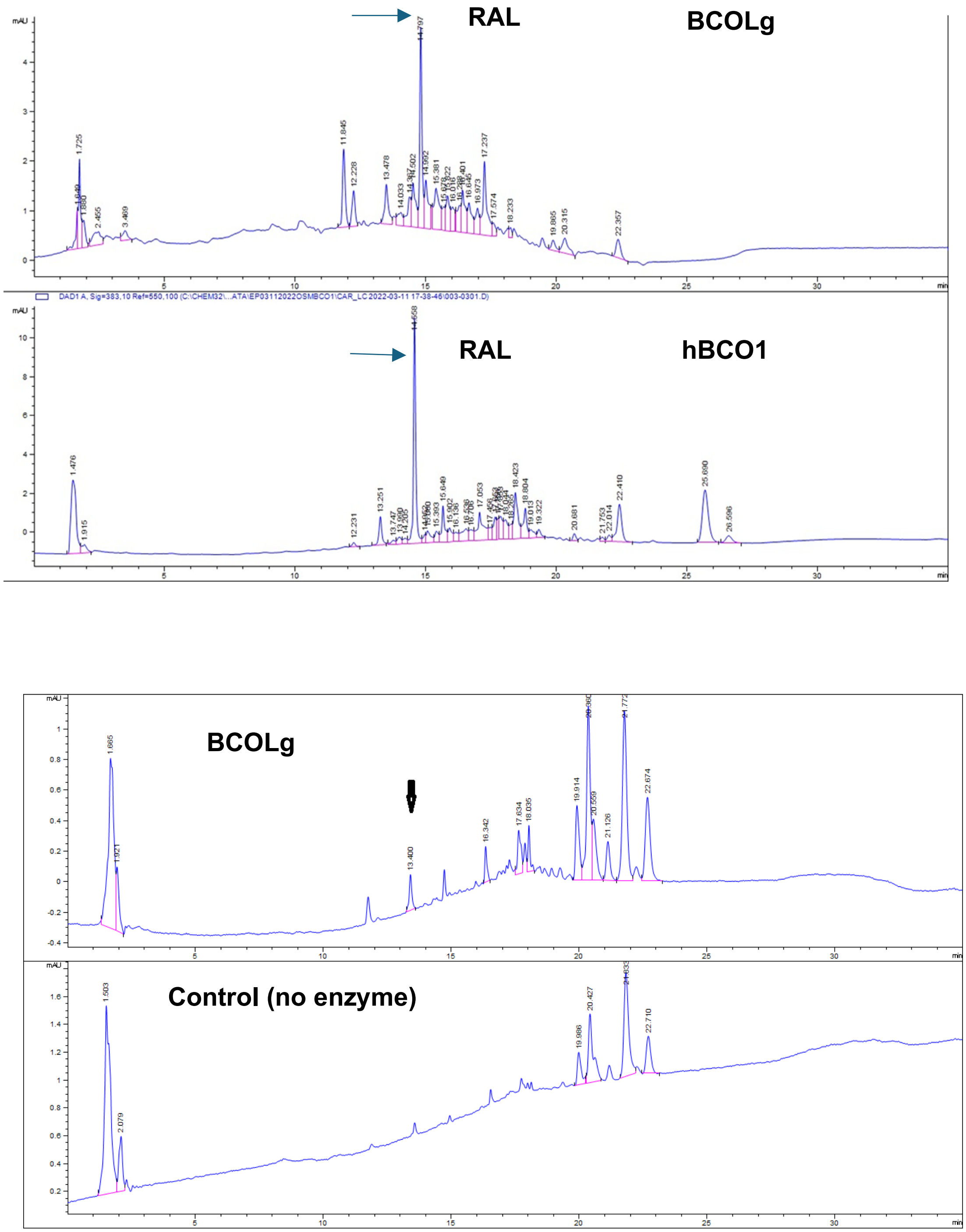

B

Figure S5. Comparison of BCOLg activity produced in Expi293 and GnTI- cells was compared to hBCOLg. (A) Comparison of BCOLg and hBCO1 activity in Expi293 cells, product of hBCO1 *al-trans* retinal is eluted at 14.6 min. (B) Activity of BCOLg produced in the glycosylation deficient cell line (GnTI-; upper panel) and no enzyme control (lower panel).

bioRxiv preprint doi: https://doi.org/10.64898/2026.10.29.762971; this version posted October 31, 2026. The copyright holder for this preprint (which was not certified by peer review) is the author/funder, who has granted bioRxiv a license to display the preprint in perpetuity. It is made available under a CC-BY 4.0 International license.

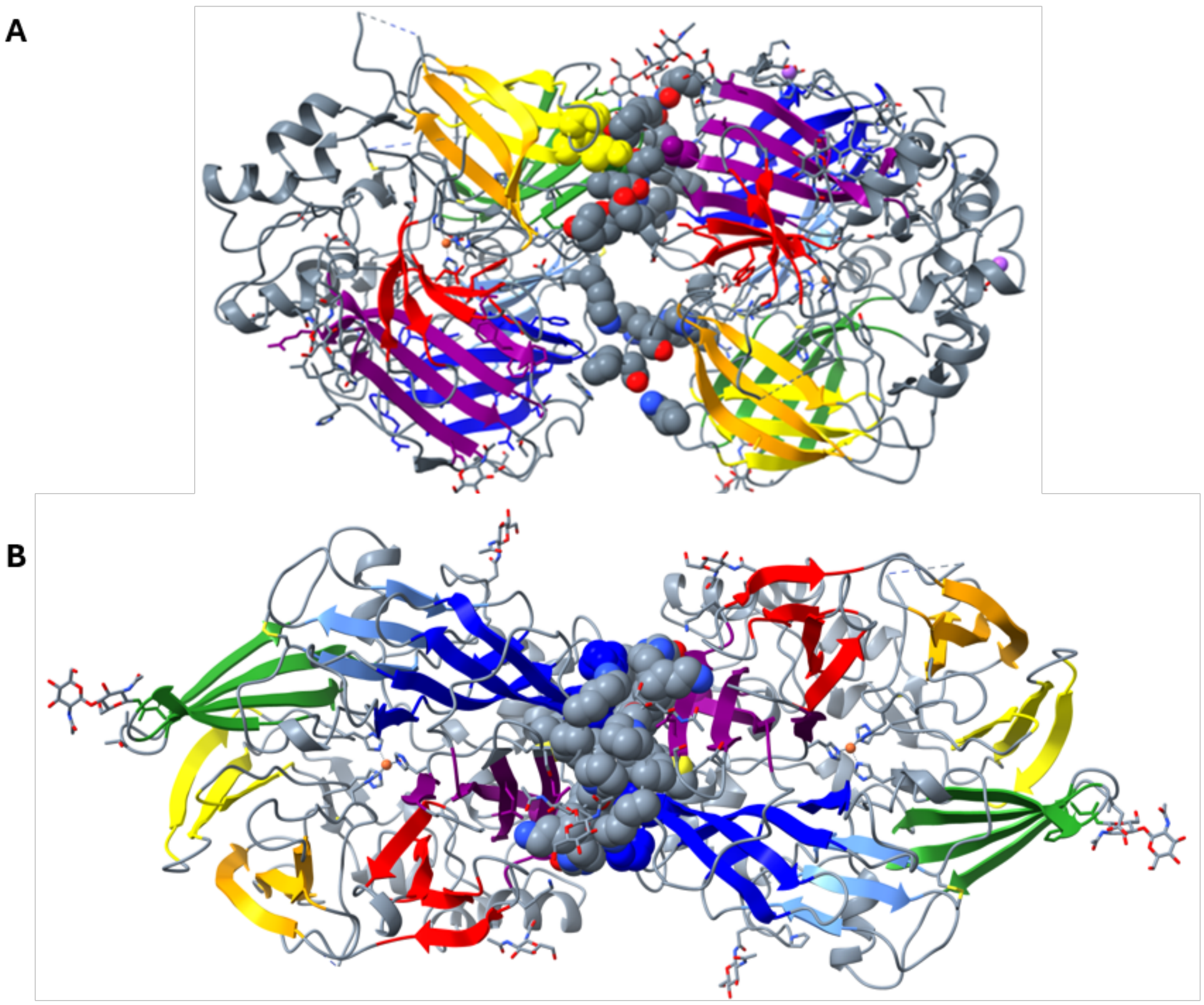

Hydrogen bonds of A/B interface

##	A	B	Dist. [Å]
1	ASP 158[OD1]	SER 216[OG]	3.77
2	SER 529[O]	ASN 270[ND2]	2.83
3	GLN 531[OE1]	TYR 271[OH]	3.67
4	ASP 158[N]	SER 216[O]	3.38
5	SER 529[OG]	GLU 334[OE1]	2.81
6	GLN 95[NE2]	GLU 337[OE2]	2.70
7	LYS 312[NZ]	SER 474[OG]	3.41

Hydrogen bonds of B/A interface

##	A	B	Dist. [Å]
1	GLU 40[O]	ARG 492[NH2]	2.76
2	HIS 42[O]	LYS 47[NZ]	3.20
3	GLY 493[O]	HIS 39[NE2]	2.76
4	ARG 492[NE]	GLU 40[O]	3.36
5	ARG 492[NH2]	GLU 40[O]	3.08
6	HIS 39[NE2]	GLY 493[O]	2.80

Figure S6. The two largest interfaces observed in the BCOLg crystal. Interface residues are shown as spheres, colored according to the blade of the seven-bladed β-propeller to which they belong. (A) The asymmetric unit contains two BCOLg molecules arranged base-to-base. The A/B interface comprises seven hydrogen bonds (listed in the table below) and buries a total surface area of 1210 Å² (600 Å² + 610 Å²). (B) The second-largest interface in the crystal is formed between molecule A and a symmetry-related molecule B (distinct from the one in the asymmetric unit) and corresponds to a side-to-side packing of two seven-bladed β-propellers that contact each other through blades VI (indigo) and VII (violet). This interface comprises six hydrogen bonds (listed in the table below) and buries a total surface area of 1480 Å² (740 Å² + 740 Å²). (C) Tables listing the hydrogen bonds at the A/B and B/A interfaces. PISA analysis was carried out with Protein interfaces, surfaces and assemblies' service PISA at the European Bioinformatics Institute as described in Methods.

bioRxiv preprint doi: https://doi.org/10.64898/2026.10.29.762971; this version posted October 31, 2026. The copyright holder for this preprint (which was not certified by peer review) is the author/funder, who has granted bioRxiv a license to display the preprint in perpetuity. It is made available under a CC-BY 4.0 International license.

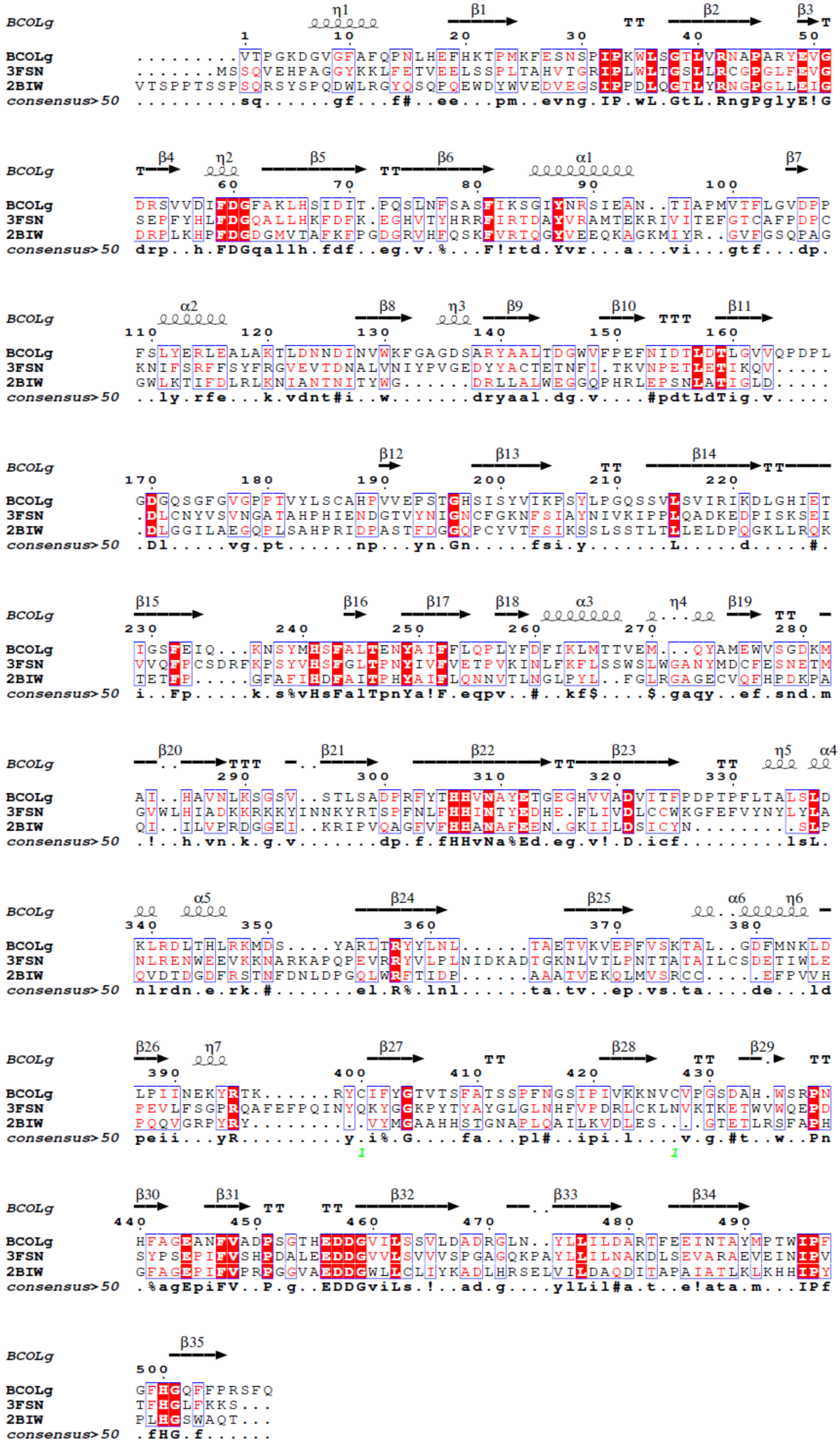

Figure S7. Sequence alignment of *Branchiostoma* BCOLg (PDB: 9PLV) with bovine RPE65 (PDB: 3FSN) and *Synechocystis* ACO (PDB: 2BIW).

bioRxiv preprint doi: https://doi.org/10.64898/2026.10.29.762971; this version posted October 31, 2026. The copyright holder for this preprint (which was not certified by peer review) is the author/funder, who has granted bioRxiv a license to display the preprint in perpetuity. It is made available under a CC-BY 4.0 International license.

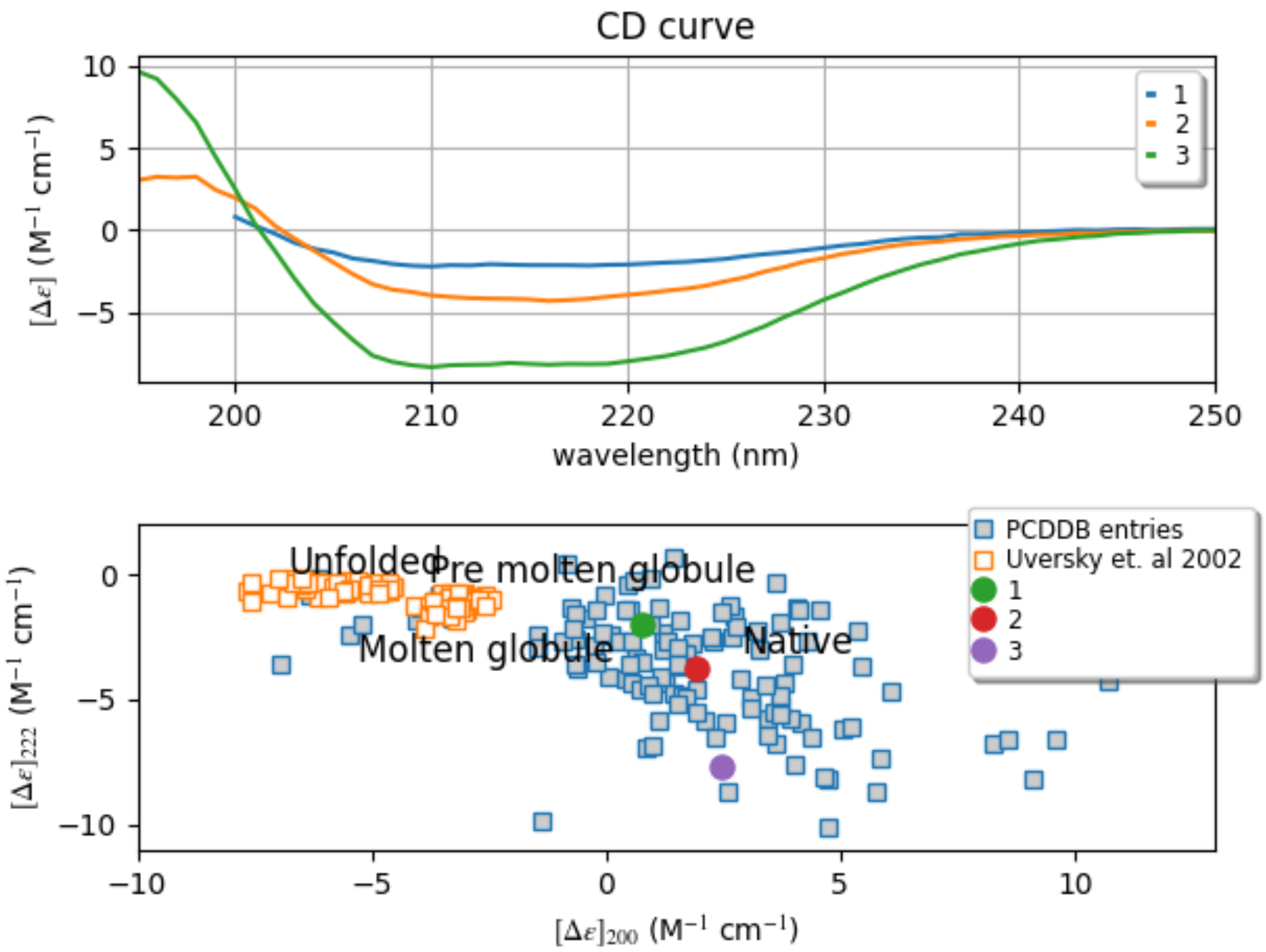

Figure S8. Far-UV circular dichroism analysis of BCOLg under different buffer conditions. Representative far-UV CD spectra (top panel) were collected between 195 and 250 nm for three BCOLg preparations. Protein 1 corresponds to BCOLg expressed in Expi293 cells and measured in Tris buffer containing glycerol. Protein 2 corresponds to the same Expi293-derived BCOLg measured in Tris buffer without glycerol following dilution. Protein 3 corresponds to BCOLg expressed in GnTI$^-$ cells and measured in Tris buffer without glycerol. Both Expi293- and GnTI$^-$-derived BCOLg proteins were purified by gel filtration in Tris buffer containing glycerol prior to CD analysis. The bottom panel shows the projection of the corresponding CD spectra onto the Protein Circular Dichroism Data Bank (PCDDB) reference map using $[\Delta\varepsilon]_{222nm}$ and $[\Delta\varepsilon]_{200nm}$ values. Blue squares represent reference proteins from the PCDDB dataset, defining regions associated with native, molten globule, pre-molten globule, and unfolded conformational states. All three BCOLg preparations cluster within the native region, indicating preservation of the overall β-sheet-rich β-propeller fold despite subtle differences in spectral intensity.

bioRxiv preprint doi: https://doi.org/10.64898/2026.10.29.762971; this version posted October 31, 2026. The copyright holder for this preprint (which was not certified by peer review) is the author/funder, who has granted bioRxiv a license to display the preprint in perpetuity. It is made available under a CC-BY 4.0 International license.

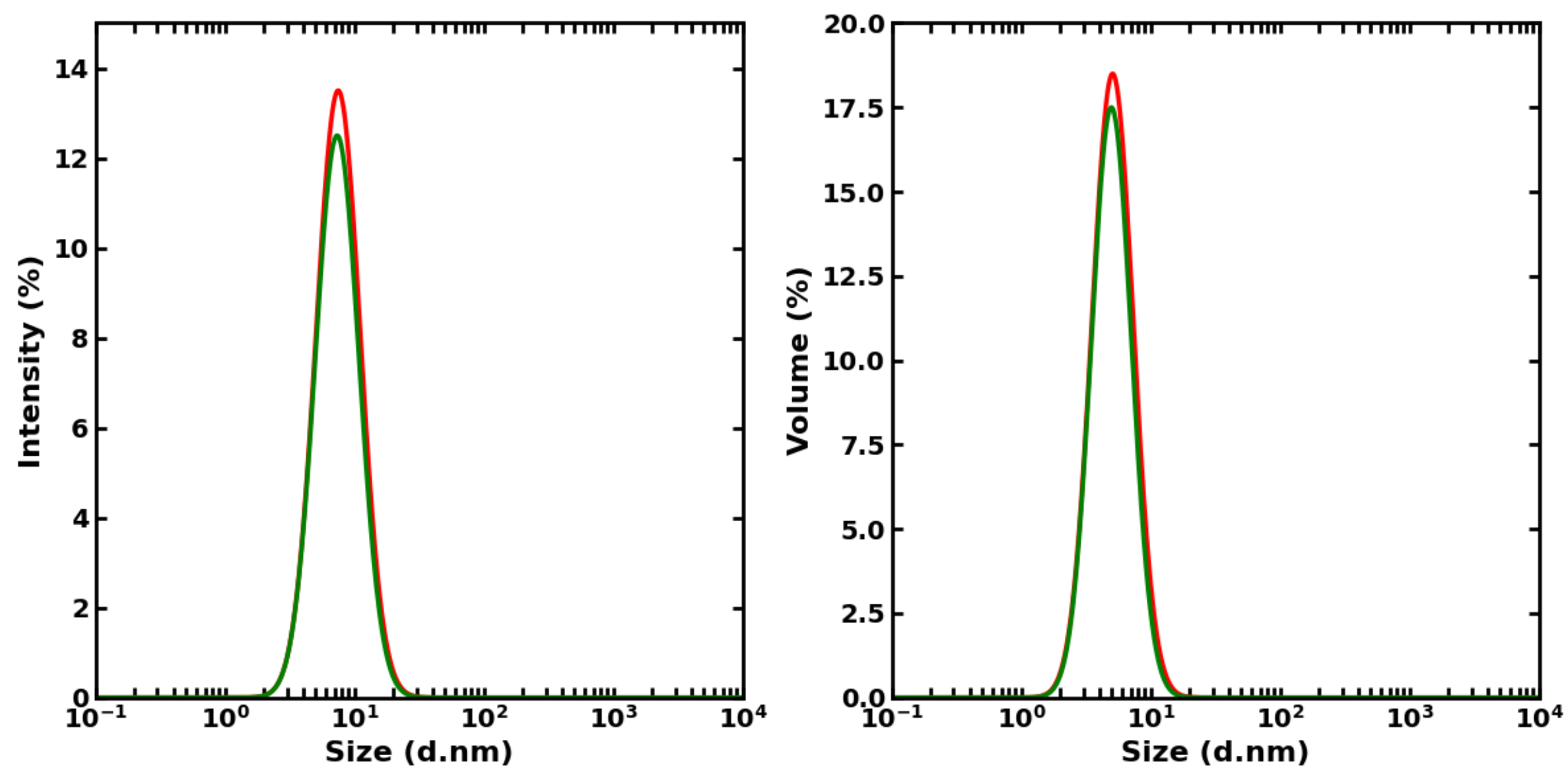

Figure S9. An example of dynamic light scattering measurement of BCOLg. BCOLg (6.8 mg/mL) used for crystallization was tested for homogeneity. PDI= 0.257, d= 6.2 ± 2.4 nm

bioRxiv preprint doi: https://doi.org/10.64898/2026.10.29.762971; this version posted October 31, 2026. The copyright holder for this preprint (which was not certified by peer review) is the author/funder, who has granted bioRxiv a license to display the preprint in perpetuity. It is made available under a CC-BY 4.0 International license.

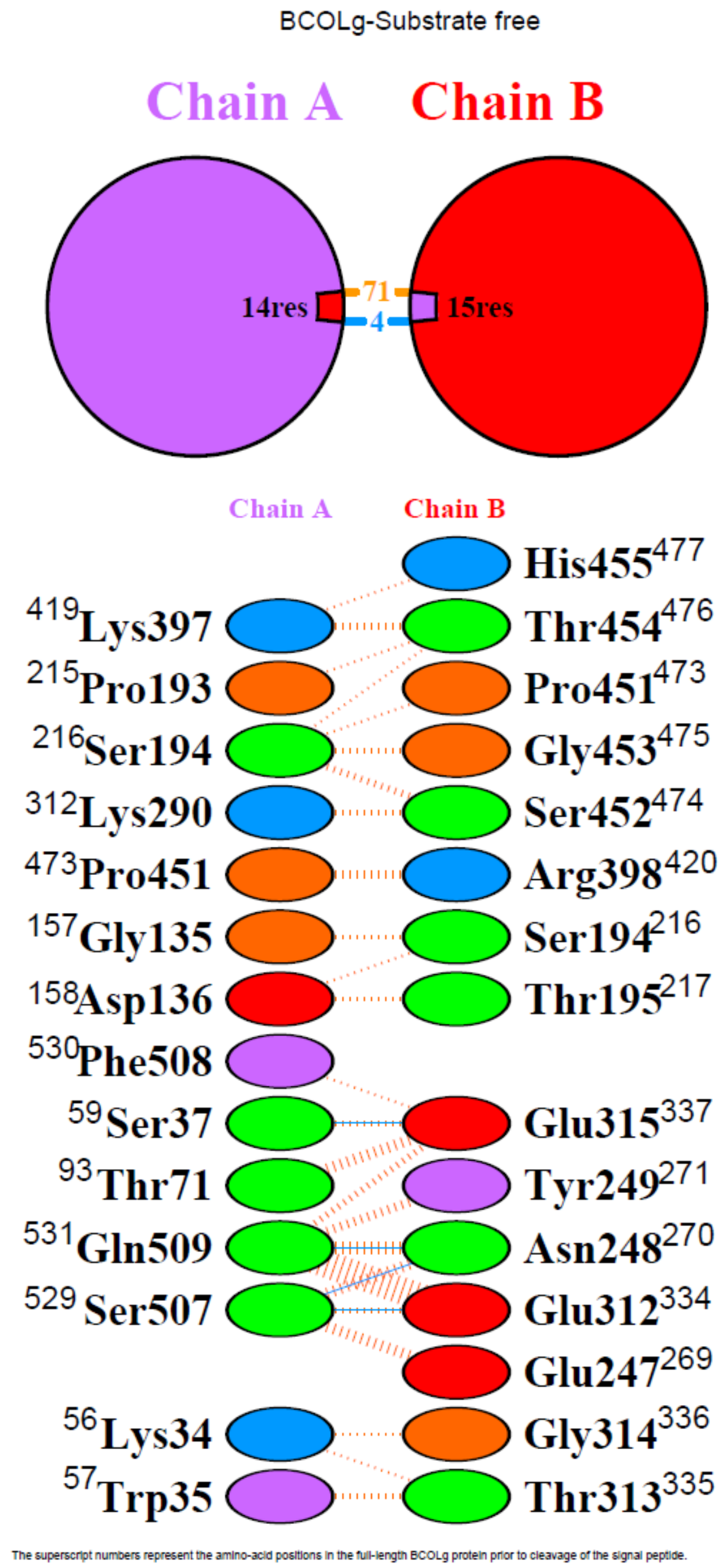

Figure S10. Interface analysis of dimeric BCOLg in the substrate-free crystal structure using PDBsum web server. Schematic representation of the interface between Chain A and Chain B in the substrate-free BCOLg crystal structure. The top panel summarizes the interface composition, showing that 14 residues from Chain A and 15 residues from Chain B contribute to the dimer interface, including multiple intermolecular contacts. The bottom panel details the specific residue–residue interactions across the interface, highlighting hydrogen bonds and other non-covalent interactions between symmetry-related chains. Residues are labeled with their positions in processed protein starting at position 23 as 1 , and superscript numbers indicate their positions in the full-length BCOLg sequence prior to signal peptide cleavage.
